# A New Scoring System to Predict Poor Clinical Outcomes in Acute Nonvariceal Upper Gastrointestinal Bleeding Patients with High-Risk Stigmata

**DOI:** 10.1155/2018/5032657

**Published:** 2018-03-12

**Authors:** Zhiyu Dong, Junwen Wang, Tingting Zhan, Haiqin Zhang, Lisha Yi, Shuchang Xu

**Affiliations:** Department of Gastroenterology, Tongji Hospital, Tongji University School of Medicine, Shanghai, China

## Abstract

**Aims:**

To explore the risk factors for rebleeding in acute nonvariceal upper gastrointestinal bleeding patients with high-risk stigmata after endoscopic hemostasis and to develop a new scoring system for them.

**Methods:**

A retrospective single-center study was conducted from January 2012 to June 2017. The logistic regression model was used to explore risk factors of poor clinical outcomes. Accuracy of new scoring systems was compared with Rockall score (RS) and Glasgow-Blatchford score (GBS) using receiver operating characteristics curve.

**Results:**

Two hundred nine patients were included. In multivariate regression analysis, systolic blood pressure, endoscopic hemostasis method, hemoglobin, blood urea nitrogen, and serum creatinine were identified as indicators for rebleeding. New scoring systems with 4 variables and 5 variables based on these 5 risk factors were chosen. The 4-variable scoring system outperformed GBS in predicting rebleeding while 5-variable scoring system outperformed RS and GBS in predicting rebleeding significantly. Score 2 was identified as the best cut-off of these 2 scoring systems.

**Conclusions:**

Systolic blood pressure, endoscopic hemostasis method, hemoglobin, blood urea nitrogen, and serum creatinine were all associated with poor clinical outcomes. The new scoring systems had greater accuracy than RS and GBS in predicting rebleeding. Further external validation should be performed to verify the results.

## 1. Introduction

Acute upper gastrointestinal bleeding (AUGIB) is a common disease with an incidence of 100 to 180 per 100,000 adults [1] while acute nonvariceal upper gastrointestinal bleeding (ANVUGIB) accounted for 80%–90% of AUGIB [2]. A systemic review showed that the incidence of rebleeding within 7 days was 13.9% while incidence of mortality was 8.6% in ANVUGIB patients [3]. Endoscopy plays an important role in diagnosis and treatment in ANVUGIB. Most ANVUGIB patients in European accepted endoscopy within 24 hours, which was considered important in management of ANVUGIB patients [4]. The endoscopic manifestation was also identified as key indicator for management of ANVUGIB [5]. Thanks to the development of endoscopic hemostasis and an increase in medical resource, the endoscopic hemostasis could be applied to the early stage of ANVUGIB once the high-risk stigmata have been found. This standpoint was recommended in many guidelines and international consensus [6–9]. Thus, the early assessment and precise management of ANVUGIB patients with high-risk stigmata after endoscopic hemostasis were also crucial.

Several scoring systems for ANVUGIB have been developed to assess the risk of patients. Rockall score (RS) is the first established and validated scoring system [10]. The system consists of multiple clinical data and endoscopic manifestation. Glasgow-Blatchford score (GBS) is the second validated scoring system [11]. The system includes UGIB symptoms and clinical data. Recently, many guidelines and international consensus recommended the prognostic scoring systems could be used for early classification of UGIB patients [6–9]. However, whether these scoring systems were suitable for the high-risk stigmata patients after endoscopic hemostasis was barely studied.

The aim of this study was to explore the risk factors for rebleeding in ANVUGIB patients with high-risk stigmata after endoscopic hemostasis. Then, we aimed to develop new scoring systems for them and compared the new systems with existing systems retrospectively.

## 2. Methods

Data were collected from consecutive patients found to have ANVUGIB with high-risk stigmata and received endoscopic hemostasis over a 5-year period, from January 2012 to June 2017, who were referred to Tongji Hospital in Shanghai, China. The ANVUGIB with high-risk stigmata was defined as spurting, gushing, oozing bleeding or nonbleeding visible vessel in the cases of peptic ulcers and spurting, gushing bleeding or nonbleeding visible vessel in the cases of other diseases.

This was a retrospective study including all medical records. Admission history, clinical and laboratory data, endoscopic manifestation, endoscopic hemostasis methods, and clinical outcomes were recorded. All patients were treated with standard supportive treatment, fluid resuscitation, and high-dose acid suppression, 80 mg pantoprazole (iv), after endoscopic hemostasis.

### 2.1. Data Collection

The following data were collected for each patient: demographic data, UGIB symptoms, history of UGIB and drug use, vital signs, laboratory results, endoscopic manifestation, endoscopic diagnosis, endoscopic hemostasis methods, and clinical outcome. The RS and GBS were calculated using the collected data for each patient.

Among them, laboratory results consist of hemoglobin (HB), blood urea nitrogen (BUN), serum creatinine (Scr), aspartate transaminase (AST), alanine aminotransferase (ALT), and blood glucose.

### 2.2. Endoscopic Procedure

All endoscopic procedures were performed by experienced endoscopist who had experience in endoscopic examination and hemostasis. Endoscopic diagnosis of bleeding was classified into spurting hemorrhage, oozing hemorrhage, and visible vessel. As for peptic ulcer bleeding, the diagnosis was followed as Forrest classification. Endoscopic hemostasis consisted of monotherapy and multiple therapies combined. The monotherapy included thermal hemostasis using argon and mechanical hemostasis using titanium clip while multiple therapies combined was defined as submucosal epinephrine injection plus thermal or mechanical hemostasis. The endoscopic hemostasis methods were chosen based on the endoscopists' judgement.

### 2.3. Clinical Outcome

The clinical outcome of the current study was defined as rebleeding after endoscopic hemostasis. Rebleeding was defined as one or more signs of bleeding after primary bleeding stopped, including fresh hematemesis or hematochezia, melena with instable vital signs, or reduction in hemoglobin levels by 3 g/dL or more.

### 2.4. Statistical Analysis

All analyses were performed using R language version 3.1.1(R Foundation for Statistical Computing, Vienna, Austria) and MedCalc version 11.4.2.0 (MedCalc software, http://www.medcalc.be).

To explore the risk of poor clinical outcomes for clinical data, the factors whose *P* < 0.1 in univariate regression analysis were included while multivariate logistic regression analysis using stepwise selection to achieve the lowest Akaike information criterion (AIC) was used to identify independent indicators of the poor clinical outcomes. Continuous variables were converted into ordinal categorical variables based on quartile.

Then, the continuous variables were also converted into unordered categorical variables based on quartile in order to determine the risk of poor clinical outcomes for each quartile of laboratory data. The binary logistic regression analysis was applied with the lowest quartiles as reference. Model 1 was unadjusted. Model 2 was adjusted for risk factors obtained in final multivariate model for each clinical outcome.

The scoring systems were made using all combinations of risk factors obtained. The cut-off of each laboratory data was identified using the intersection of the best 5 cut-offs for each clinical outcome. The best cut-off was based on Youden's index.

The accuracy of the scoring systems to predict clinical outcomes was evaluated by receiver operating characteristics curves (ROC) with 95% confidence intervals. The areas under ROC curves (AUC) were compared using chi-square tests according to the method described by Delong et al. [12].

All reported *P* values were two-sided with *P* < 0.05 defined as statistical significance.

## 3. Results

### 3.1. Patient Characteristics

A total of 209 patients were included in the study.  Table 1 describes the demographics and clinical and endoscopic characteristics of these patients. One hundred seventy*-*three males and 36 females were studied, whose median age was 58. For the clinical outcomes, 38 patients bled one or more times after primary bleeding stopped and 44 patients had requirement of blood transfusion. Duodenal ulcer and gastric ulcer were the main causes of ANVUGIB. For endoscopic hemostasis, 172 patients accepted monotherapy consisting of 101 mechanical hemostasis and 71 thermal hemostasis. Thirty*-*seven patients received multiple hemostasis combined.

### 3.2. Logistic Regression Analysis for Rebleeding

Table 2 demonstrates the differences of risk factors between the rebleeding group and no rebleeding group by giving statistical significance using logistic regression model. In multivariate regression analysis, systolic blood pressure (SBP) (<90 mmHg), endoscopic hemostasis method, HB, BUN, and Scr were included in final regression model. But only SBP, endoscopic hemostasis method, HB, and Scr were the independent indicators for rebleeding.

As demonstrated in Table 3, the results of logistic regression analysis revealed the risk of HB, BUN, and Scr, which were considered as unordered categorical variables. After adjusted for SBP, endoscopic hemostasis method, and other included laboratory data, the results showed increased risk of rebleeding with HB and Scr ascending. The Q4 of HB and Scr was significantly associated with higher hazard compared to those in the lowest quartile. The Q3 of BUN has the highest hazard in the 4 quartiles. It was inverted U-shaped relation between BUN and risk of rebleeding. Moreover, there is almost no difference between the hazard of the Q2 (range 6.6~10 mmol/L) and Q1 (range < 6.6 mmol/L). Despite no statistically significant, the Q3 and Q4 have higher hazard compared to Q1 and Q2 in multivariate analysis while the Q4 of BUN was significantly associated with higher hazard compared to the lowest quartile in univariate analysis. Thus, HB, BUN, and Scr were all the key predictors for rebleeding.

### 3.3. Development of the New Scoring Systems

Totally, 5 factors were included in our new scoring systems based on the final multivariate regression model. The cut-off of continuous variables including HB, BUN, and Scr was identified using the intersection of the best 5 cut-offs based on Youden's index for each clinical outcome (Table 4). In order to make the cut-off well remembered and meet the clinical practicality, the cut-offs of BUN and Scr were set as 9.5 mmol/L and 100 *μ*mol/L, respectively. As for HB, the best 5 cut-offs for rebleeding were around 75 g/L or 85 g/L. Calculating the sensitivity and specificity of both cut-offs, the results showed too low sensitivity in cut-off 75 g/L (rebleeding sensitivity/specificity 0.47/0.86) and cut-off 85 g/L has better result (rebleeding sensitivity/specificity 0.58/0.75). Thus, HB < 85 g/L was finally selected as cut-off value. All of these 5 factors were weighted equally for simplicity.

A total of 31 combinations were created and AUC of each was calculated. Among them, the set of variables (from 1 to 5) that yielded the highest AUC value is shown in Table 5. Four-variable (Scr > 100 *μ*mol/L; BUN > 9.5 mmol/L; HB < 85 g/L; monotherapy) and 5-variable (Scr > 100 *μ*mol/L; BUN > 9.5 mmol/L; HB < 85 g/L; monotherapy; SBP < 90 mmHg) scoring systems were the best score for predicting rebleeding. The cut-off 2 in 4-variable scoring system provided the high sensitivity (rebleeding 94.7%) and patients with score 0 had no risk for rebleeding. The cut-off 2 in 5-variable scoring system provided high sensitivity (94.7%) while patients with score 0 had no risk for rebleeding either.

### 3.4. Comparison of New Scoring Systems with RS and GBS

In predicting rebleeding, 4-variable scoring system (AUC 0.78 (0.71–0.85)) performed as well as RS (AUC 0.70 (0.61–0.79)) and outperformed GBS (AUC 0.71 (0.62–0.8)) significantly (*P* = 0.049) while 5-variable scoring system (AUC 0.79 (0.72–0.86)) outperformed both RS (*P* = 0.046) and GBS (*P* = 0.021) significantly (Table 6).

## 4. Discussion

ANVUGIB is a common digestive system disease and a frequent cause of poor clinical outcome. Recently, international consensus recommended the prognostic scoring systems should be used for early assessment of ANVUGIB patients [6–9]. Early assessment of patients at high risk can improve the efficiency of treatment and clinical outcomes for patients. For example, better allocation of medical resources could be administered after early identification of patients. A systemic review evaluating the accuracy of RS and GBS demonstrated that GBS excelled RS in identifying patients who did not require any intervention [13]. Recently, a multicenter prospective cohort study enrolling 1584 AUGIB patients illustrated the value of GBS in predicting hospital-based intervention and the superiority of RS in predicting death [14]. As the development of endoscopy, more patients could accept endoscopy within 24 hours and the endoscopic manifestation was considered as a key indicator for management. The endoscopic hemostasis was superior to pharmacotherapy in patients with high-risk stigmata [15]. Thus, the patients with high-risk stigmata found in endoscopy could receive hemostasis simultaneously. The improved clinical pathway decreased the risk of these patients, but the existing scoring systems were not updated to fit changed medical condition and whether the existing scoring systems are suitable for high-risk stigmata patients after endoscopic hemostasis was barely studied. Thus, it is necessary to construct new scoring systems with higher accuracy and better performance for ANVUGIB patients with high-risk stigmata after endoscopic hemostasis.

To make the cut-off well remembered and meet the clinical practicality, the cut-off of HB, BUN, and Scr was set as 85 g/L, 9.5 mmol/L, and 100 *μ*mol/L. HB, BUN, and Scr were all considered as crucial indicators for prognosis. Among them, HB and BUN were included in GBS, but the complexity of calculating made doctors seldom used this risk score. Additionally, there was not obvious difference between BUN (6.6~10 mmol/L) group and BUN (≤6.5 mmol/L) group in predicting rebleeding, but BUN (> 10 mmol/L) group had increased hazard of rebleeding. Moreover, Scr was not included in GBS, but it is really a key indicator for poor clinical outcomes. As an ordinal categorical variable, Scr was independent predictor for rebleeding in multivariate regression. As an unordered categorical variable, Scr (≥94 *μ*mol/L) group had several risk of poor clinical outcomes compared with Scr (<94 *μ*mol/L) group. After integrated analysis of logistic regression model and the best 5 cut-offs of each laboratory index, we adjusted the cut-off of these 3 laboratory data to most fit and easy-remembered status, which were different from the cut-off in GBS. SBPs were also indicators of patients' condition. A previous trial demonstrated that the shock index, HR/SBP, was independent predictor for high-risk stigmata and endoscopic intervention and the simple score consisting of shock index, BUN/Scr and “no daily use PPI one week before examination”, was superior to GBS [16]. However, the shock index was not included in final multivariate regression model for rebleeding while SBPs were independent predictors for rebleeding in our results. Then, the frequently used cut-off “SBP < 90 mmHg” was set. Recently, the international consensus and guidelines demonstrated the mechanical, thermal hemostasis and epinephrine plus any second hemostasis were all effective methods for achieving hemostasis [9]. In our results, monotherapy was found to be independent risk factor for both rebleeding. Thus, “Monotherapy” was also set as one factor in our new scoring system. Finally, 4-variable and 5-variable scoring systems were identified.

The 4-variable scoring system, which consists of 4 variables, namely, “HB < 85 g/L,” “BUN > 9.5 mmol/L,” “Scr > 100 *μ*mol/L,” and “Monotherapy”, performed as well as RS and outperformed GBS significantly in predicting rebleeding. Moreover, the cut-off 2 for this scoring system provided very high sensitivity and good specificity in predicting rebleeding. If the intensive treatment was only performed on scores of 2, 3, and 4, almost all of high-risk patients could receive special care to decrease the risk of rebleeding while about 45% clinical resource could be saved. The 5-variable scoring systems, which comprise of 5 variables, “SBP < 90 mmHg,” “HB < 85 g/L,” “BUN > 9.5 mmol/L,” “Scr > 100 *μ*mol/L,” and “Monotherapy”, outperformed RS and GBS in predicting rebleeding. The cut-off 2 provided very high sensitivity in predicting rebleeding, but the specificity was lower than that of 4-variable scoring system. In these 2 scoring systems, the patients with a score of zero could be normally managed as they were less likely to suffer rebleeding after endoscopic hemostasis.

As we know, the difficulty of calculating and distrust led to the factor that doctors were unwilling to use risk score the guidelines recommended for management of UGIB patients. Our simple score, no matter 4 variables or 5 variables were reliable, was easy-remembered and easy-calculated.

Despite the good performance of our new scoring systems, there were several limitations. One limitation was the small sample size. The small sample size makes it hard to draw a firm conclusion while many risk factors for clinical outcomes might be covered up. Moreover, the comparison of different endoscopic hemostasis methods such as thermal hemostasis versus multiple therapies combined and mechanical hemostasis versus multiple therapies combined was hard to achieve due to the small sample size. Another limitation is that our study is retrospective single-center clinical trial, which limits its reliability and generalizability potentially.

In summary, SBP, HB, BUN, Scr, and endoscopic hemostasis methods were indicators for rebleeding in ANVUGIB patients with high-risk stigmata after endoscopic hemostasis. The 4-variable scoring systems we made were greater than GBS in predicting rebleeding significantly and 5-variable scoring systems we made outperformed RS and GBS in predicting rebleeding. However, larger multicenter prospective studies are needed to validate these conclusions and verify the new scoring systems thresholds that might be suitable for clinical decisions in the future.

## Figures and Tables

**Table 1:** The characteristics of acute nonvariceal upper gastrointestinal bleeding patients enrolled.

	Number of patients
Clinical characteristics	
Age: median(IQR)^a^	58 (42, 66)
Gender	
Male	173
Female	36
UGIB symptom(s)^b^	
Melena	181
Hematemesis	107
Syncope	23
Outcomes	
Rebleeding	38
Blood transfusion	44
Further intervention	15
Mortality	9
Endoscopic characteristics	
Manifestation	
Gastric ulcer	57
Duodenal ulcer	101
Anastamotic ulcer	3
Esophageal ulcer	3
Gastritis	5
Duodenitis	10
Anastamotic bleeding	10
Mallory-Weiss	16
Gastric vascular ectasia	1
Malignancy	3
Endoscopic hemostasis	
Monotherapy	172
Multiple therapies combined	37

^a^IQR: interquartile range. ^b^Some patients presented with more than one symptom.

**Table 2:** Univariate and multivariate analysis between rebleeding case and no rebleeding case.

	Rebleeding case (*n* = 38)	No rebleeding case (*n* = 171)	*P*1 value	*P*2 value
Age (>60), number (%)	17 (44.7%)	66 (38.6%)	0.485	
Sex (male), number (%)	30 (78.9%)	143 (83.6%)	0.491	
HR (>100 beats/min), number (%)	12 (31.6%)	27 (15.8%)	0.027	
SBP (<90 mmHg), number (%)	5 (13.2%)	6 (3.5%)	0.025	0.016
Alcohol (yes), number (%)	9 (23.7%)	31 (18.1%)	0.433	
Smoke (yes), number (%)	14 (36.8%)	50 (29.2%)	0.359	
UGIB history (yes), number (%)	8 (21.1%)	40 (23.4%)	0.757	
Multiple therapies, number (%)	3 (7.9%)	34 (19.9%)	0.092	0.015
HR/SBP, mean (SD)	0.89 (0.38)	0.73 (0.21)	0.023	
HB, mean (SD), g/L	86.82 (26.94)	105.25 (26.47)	0.001	0.002
BUN, mean (SD), mmol/L	13.53 (6.92)	10.55 (5.77)	0.004	0.115
Creatinine, mean (SD), *μ*mol/L	103.55 (46.49)	82.08 (49.39)	0.001	0.004
Glucose, mean (SD), mmol/L	8.09 (3.44)	7.93 (3.97)	0.188	
ALT, mean (SD), U/L	41.13 (70.54)	28.71 (22.28)	0.268	
AST, mean (SD), U/L	42.37 (84.1)	24.73 (26.97)	0.693	

HR: heart rate; SBP: systolic blood pressure; HB: hemoglobin; BUN: blood urea nitrogen; AST: aspartate transaminase; ALT: alanine aminotransferase; multiple therapies was defined as submucosal epinephrine injection plus thermal or mechanical hemostasis.

**Table 3:** The risk of rebleeding for each quartile of laboratory data.

	Q1	Q2	Q3	Q4	*P* for trend
HB (g/L)	*n* = 52	*n* = 52	*n* = 49	*n* = 56	
Range	121~160	103~120	81~102	42~80	
Model 1	Ref.	1.87 (0.53–7.53)	2.34 (0.69–9.3)	6.16 (2.11–22.65)	
*P* values		0.344	0.189	0.002	0.001
Model 2	Ref.	1.42 (0.35–6.19)	2.55 (0.71–10.51)	5.6 (1.73–22.2)	
*P* values		0.623	0.163	0.007	0.002
BUN (mmol/L)	*n* = 53	*n* = 54	*n* = 53	*n* = 49	
Range	2.85~6.5	6.6~10	10.1~14.7	14.8~42.8	
Model 1	Ref.	1.43 (0.43–5.13)	2.51 (0.84–8.52)	4.24 (1.48–14.04)	
*P* values		0.564	0.111	0.01	0.004
Model 2	Ref.	1.08 (0.29–4.2)	2.56 (0.77–9.54)	2.08 (0.62–7.74)	
*P* values		0.911	0.136	0.246	0.115
Scr (*μ*mol/L)	*n* = 55	*n* = 55	*n* = 48	*n* = 51	
Range	37~66	67~76	77~93	94~669	
Model 1	Ref.	1.7 (0.53–5.98)	1.71 (0.51–6.15)	5.45 (1.96–17.83)	
*P* values		0.379	0.39	0.002	0.001
Model 2	Ref.	1.83 (0.53–6.95)	1.97 (0.54–7.76)	5.94 (1.86–22.13)	
*P* values		0.351	0.31	0.004	0.004

HB: hemoglobin; BUN: blood urea nitrogen; Scr: serum creatinine; Model 1: crude, no adjustment; Model 2: adjusting for risk factors in final multivariate model; Ref: reference.

**Table 4:** The best 5 cut-offs of each laboratory data for predicting rebleeding.

	5th	4th	3rd	2nd	1st
HB (g/L)	74	84	77	86	73
Youden's index	0.333	0.333	0.336	0.342	0.345
BUN (mmol/L)	11.9	9.7	9.3	9.8	9.4
Youden's index	0.254	0.257	0.260	0.263	0.272
Scr (*μ*mol/L)	94	95	99	100	102
Youden's index	0.281	0.287	0.287	0.292	0.295

HB: hemoglobin; BUN: blood urea nitrogen; Scr: serum creatinine; Youden's index: sensitivity + specificity − 1.

**Table 5:** The combination of variables that yielded the highest AUC value with sensitivity and specificity.

Number of variable	Variables for rebleeding	AUC	Cut-off	Sensitivity	Specificity
1	HB < 85 g/L	0.67	1	57.9	75.4
2	Scr > 100 *μ*mol/L	0.74	1	76.3	71.3
	HB < 85 g/L		2	21.1	94.2
3	Scr > 100 *μ*mol/L	0.76	1	100	10.5
	HB < 85 g/L		2	71.1	77.8
	Monotherapy		3	18.4	95.3
4	Scr > 100 *μ*mol/L	0.78	1	100	7.6
	BUN > 9.5 mmol/L		2	94.7	43.9
	HB < 85 g/L		3	52.6	88.3
	Monotherapy		4	15.8	95.3
5	Scr > 100 *μ*mol/L	0.79	1	100	7.6
	BUN > 9.5 mmol/L		2	94.7	42.7
	HB < 85 g/L		3	57.9	86.0
	Monotherapy		4	23.7	95.3
	SBP < 90 mmHg		5	—	—

HB: hemoglobin; BUN: blood urea nitrogen; Scr: serum creatinine; SBP: systolic blood pressure; monotherapy was defined as mechanical or thermal hemostasis.

**Table 6:** Accuracy of new scoring systems compared with existing scoring systems.

	Area under the receiver-operator curves (AUC)	95% confidence interval	Comparison to new score (*P* value)
4 variables			
New score	0.784	0.71–0.85	—
Rockall score	0.699	0.61–0.79	0.083
GBS full	0.708	0.62–0.80	0.049
5 variables			
New score	0.792	0.72–0.86	—
Rockall score	0.699	0.61–0.79	0.046
GBS full	0.708	0.62–0.80	0.021

GBS: Glasgow-Blatchford score.
